# Study on the Developmental Differences Between Female and Male Early Embryos in Cattle In Vivo

**DOI:** 10.3390/genes16121485

**Published:** 2025-12-11

**Authors:** Jie Wang, Fei Huang, Di Fang, Peng Niu, Jie-Ru Wang, Qing-Hua Gao

**Affiliations:** 1 College of Animal Science and Technology, Tarim University, Alar 843300, China; wangjie1235@126.com (J.W.); 107572022315@stumail.taru.edu.cn (F.H.); 120230093@taru.edu.cn (D.F.); 107572021314@stumail.taru.edu.cn (J.-R.W.); 2 Key Laboratory of Tarim Animal Husbandry Science and Technology, Xinjiang Production & Construction Corps, Alar 843300, China; 10757203053@taru.edu.cn

**Keywords:** in vivo bovine embryo, in vivo fertilization, embryonic development, sex differences

## Abstract

Background: The developmental differences between female and male early embryos regarding sex development remain a topic of controversy. Objectives: This study aims to investigate whether there are significant developmental differences between female and male bovine embryos during in vivo development. Methods: The CIDR + FSH + PGF2α + GnRH method was employed to induce superovulation in 20 donor cows. Subsequently, artificial insemination was performed on the donor cows using high-purity X and Y frozen semen, with 10 cows receiving each type of semen. Seven days later, the embryos were flushed from the donor cows. The flushed embryos underwent embryonic sex determination, followed by immunofluorescence analysis to observe proliferation and apoptosis, and finally, RT-PCR was conducted to detect genes associated with proliferation and apoptosis. Results: The results indicated that the sex ratio of embryos obtained through artificial insemination using X/Y semen did not significantly differ based on semen purity (*p* ≥ 0.05). However, the fluorescence intensity of apoptotic cells in the X-BL group was significantly higher than that in the Y-BL group (*p* < 0.05). Conversely, the fluorescence intensity of proliferating cells in the X-BL group was significantly lower than that in the Y-BL group (*p* < 0.05). Furthermore, the expression levels of apoptosis-related genes in the X-BL group were significantly higher compared to the Y-BL group (*p* < 0.05), while the expression levels of proliferation-related genes in the X-BL group were significantly lower than those in the Y-BL group (*p* < 0.01). Conclusions: The above results indicate that during in vivo development of bovine early embryos, male embryos develop at a faster rate than female embryos.

## 1. Introduction

The developmental differences between early embryos (blastocysts) of different sexes have long been a focal point of scientific inquiry [1,2]. Reproductive biologists continue to engage in debates regarding the sex-based differences in the growth of male and female embryos [3,4]. Numerous studies have underscored the sex-specific variations in the growth and developmental dynamics of mammalian embryos during the pre-implantation stage [5]. However, a definitive answer remains elusive to date.

The rapid growth of embryos prior to implantation is a key indicator of embryo health [6]. These rapidly developing embryos are prioritized in transplantation, cryopreservation, and commercial breeding practices [7,8]. However, some studies suggest that male embryos develop at a faster rate than female embryos [9,10]. Research conducted by Samer Alfarawati et al. indicates that sex is associated with blastocyst grading, revealing that male embryos develop significantly faster than their female counterparts [11,12]. Additionally, findings from Tapinder Sidrat et al. demonstrate that on day seven, 57.8% of Y-sperm sorted embryos in in vitro culture reached the expanded blastocyst (BL) stage, in contrast to only 25% in the X-sperm sorted group [13]. Conversely, some studies have reported no significant differences in the development rates of female and male embryos [14,15,16]. Furthermore, existing research on the developmental differences between male and female embryos predominantly focuses on samples obtained through in vitro culture, with no clear data currently available to support differences in early in vivo embryonic development between the sexes [17].

To assess the differences in developmental dynamics between male and female embryos without the interference of the culture medium environment or sperm conditions, this study employed the CIDR + FSH + PGF2α + GnRH method to induce superovulation in 20 donor cows. High-purity X and Y frozen semen were then utilized for the artificial insemination of the donor cows, with 10 cows receiving each type of semen. Seven days later, the embryos were flushed from the donor cows. The flushed embryos underwent sex identification, followed by immunofluorescence observation of proliferation and apoptosis, as well as RT-PCR detection of related genes. The ultimate aim was to determine whether differences exist in the developmental dynamics of male and female early embryos in vivo.

## 2. Materials and Methods

### 2.1. Ethics Statement

All animal experiments were conducted in compliance with the “Regulations and Guidelines for the Management of Experimental Animals” established by the Ministry of Science and Technology (Beijing, China, 2020 revision). This study received approval from the Institutional Animal Care and Use Committee of Tarim University, Xinjiang, China (protocol code DWBH20220101; approval date: 1 January 2022).

### 2.2. Selection of Donor Cows

This study involved 20 Simmental cows, each having calved 2 to 3 times, 4–5 years old, exhibiting normal reproductive tracts and ovarian functions, with an average weight of 480 ± 50 kg and a body condition score of 3.5 ± 0.5. During the trial, the superovulated donor cows were fed a total mixed ration (TMR) and were provided with ample clean drinking water.

### 2.3. Selection of a Superovulation Treatment Plan

**Figure 1:**
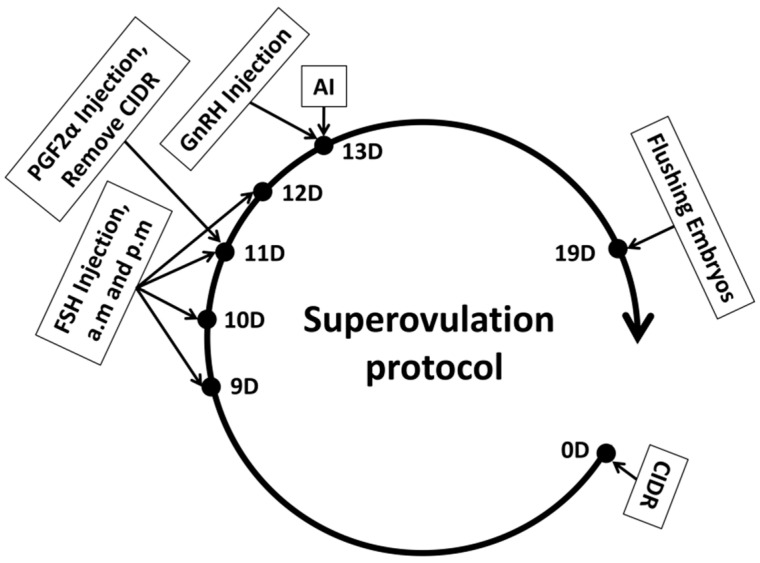
Flowchart of the superovulation protocol for donor cows.

Superovulation Treatment: This study employed the CIDR + FSH + PGF2α + GnRH protocol for superovulation in donor cows [18]. Follicle-stimulating hormone (FSH) is essential for stimulating the ovaries to release multiple oocytes within a single cycle. The experimental protocol is illustrated in Figure 1. On Day 0, a Controlled Internal Drug Release (CIDR) device containing progesterone was inserted intravaginally. From Day 9 to Day 12, FSH preparation (700 IU/vial, 20 mL/vial, Vetoquinol™, Brisbane, Australia) was administered intramuscularly twice daily at 12 h intervals, following a decreasing dosage regimen: Day 9: 140 IU (2 doses); Day 10: 105 IU (2 doses); Day 11: 70 IU (2 doses); Day 12: 35 IU (2 doses). On Day 11, 30 µg of PGF2α (10 vials/box, 0.2 mg/vial, NSHF™, Beijing, China) was injected in the morning after FSH administration, and the CIDR was removed in the afternoon. On Day 13, 30 µg of GnRH (100 µg/vial, NSHF™, China) was injected, after which follicular development was monitored through rectal examination and B-ultrasound.

### 2.4. Artificial Insemination

Artificial insemination was conducted using commercially available high-purity sorted frozen semen of X and Y (same batch; IDs: 41119908X, 41119908Y). X frozen semen was utilized for 10 donor cows, while Y frozen semen was employed for the remaining 10. Insemination took place 8 to 12 h after the cows exhibited estrus, with subsequent inseminations occurring every 12 h. A third insemination was performed if estrus persisted. On Day 19, rectal and B-ultrasound examinations were carried out to detect and record the number of corpora lutea formed in the ovaries, followed by non-surgical embryo collection.

### 2.5. Non-Surgical Embryo Flushing Was Performed as Follows

Initially, the donor cow was administered 150 µg of lidocaine hydrochloride for local anesthesia. After ensuring adequate disinfection, a dilator rod was inserted into the cervix, followed by the placement of an embryo collection catheter into the uterus. The catheter balloon was inflated to occlude the uterine horn, adjusted according to its size. A syringe-type embryo flushing catheter was employed to draw phosphate-buffered saline (PBS) using a 50 mL syringe. PBS was then infused through the catheter into the uterine horn, which was gently massaged via rectal manipulation, allowing the PBS to be subsequently aspirated into an embryo collection cup. Each uterine horn was flushed with 500 mL of PBS. The collected embryos were evaluated under a microscope, with Grade 1 and Grade 2 blastocysts defined as viable embryos in this study [19].

### 2.6. Identification of Embryonic Gender

In this study, embryo recovery was conducted on the seventh day following artificial insemination, with the embryos expected to be in the blastocyst stage. Female blastocysts are designated as X-BL, while male blastocysts are designated as Y-BL. The blastocysts were washed in phosphate-buffered saline (PBS) before being individually placed into 0.2 mL nuclease-free centrifuge tubes. To each tube, 5 μL of a genome extraction solution (50 mmol/L Tris-HCl, pH 8.0, 0.5% Triton X-100, 1 mg/mL Proteinase K) was added. Each test tube contained one blastocyst, followed by lysis. The 5 μL of genome extraction solution obtained post-lysis was used as the template for polymerase chain reaction (PCR). Primers targeting the amelogenin (AMELY) gene, as detailed in Table 1, were designed to facilitate PCR-based gender determination of the individual embryos. Primer synthesis was completed by Shanghai Sangon Biotech Co., Ltd., Shanghai, China. The working concentration of the primers was 10 ng/μL. For the second round of PCR, 1 μL of the product from the first round served as the template. The nested PCR procedures for both rounds were identical, ensuring consistency in the amplification process. Subsequently, the PCR products were subjected to 2% agarose gel electrophoresis at 100 V for 20 min, followed by visualization and photography using a gel imaging system.

### 2.7. Detection of Proliferation and Apoptosis in Embryos

Both proliferation and apoptosis assays were performed using Grade 1 blastocysts. The proliferation rates of the blastocysts were evaluated using the BrdU assay. The embryos that were collected underwent a washing process with PBS buffer, repeated three times, and were subsequently fixed in a solution of 4% paraformaldehyde for a duration of 30 min. Following this, the embryos experienced an additional three washes with PBS before being exposed to 0.5% acid Tyrode’s solution for 5 min. Afterward, the embryos were subjected to three more washes with PBS, then transferred to a solution of 0.5% Triton X-100 for 30 min, which was again followed by three washes with PBS. Subsequently, an overnight incubation at 4 °C with a BrdU antibody (diluted 1:100; B100-1 µL, Sigma, Burlington, MA, USA) was performed. After three washes in PBS, the embryos were treated with FITC-conjugated goat anti-rabbit IgG (diluted 1:1000; ab6939, Abcam, Shanghai, China) at room temperature for a period of 2 h. Following thorough PBS washes, embryos were counterstained with DAPI for 5 min at room temperature. Once three additional washes with PBS were completed, individual embryos were chosen and mounted on glass slides using 2 µL of anti-fluorescence quenching mounting medium (P0131; Beyotime Biotechnology, Shanghai, China). The analysis of cell proliferation rates was carried out using a confocal laser scanning microscope (FV1000, Olympus Fluoview system, Olympus Corp, Tokyo, Japan). The proliferation index for each embryo was determined by dividing the count of BrdU-positive nuclei by the overall number of nuclei within the embryo. To evaluate levels of apoptosis in the embryos, a process of washing them three times in PBS buffer was performed, followed by fixation in a 4% paraformaldehyde solution for 30 min. After three PBS washes, embryos were treated with 0.5% acid Tyrode’s solution for 5 min, which was then followed by three additional washes with PBS and a 30-min incubation in 0.5% Triton X-100. The permeabilized embryos then underwent an overnight incubation in TUNEL solution in the dark at 4 °C. Then, stained embryos were washed and subsequently incubated with DAPI for 5 min. After completing three more washes with PBS, individual embryos were selected and mounted onto slides using 2 µL of anti-fluorescence quenching medium. Imaging was performed with the confocal laser scanning microscope (FV1000, Olympus Fluoview system). The average percentage of TUNEL-positive nuclei per embryo was determined by dividing the number of positive nuclei by the total cell count (stained with DAPI). The fluorescence wavelength of DAPI is 340 nm; that of Tunel ranges from 450 nm to 500 nm; and that of BrdU ranges from 450 nm to 655 nm. At least five randomly selected blastocysts were observed, and each sample was analyzed in triplicate.

### 2.8. Embryo RNA Extraction and Detection of Proliferation and Apoptosis-Related Genes

Total RNA was isolated from individual embryos utilizing an ultramicro RNA extraction kit (DP420; Tiangen Biotech (Beijing) Co., Ltd., Beijing, China). This isolated RNA underwent reverse transcription into cDNA with the help of a reverse transcription kit (KR116-02; Tiangen Biotech (Beijing) Co., Ltd., China). From a single blastocyst, cDNA was reverse-transcribed and amplified through the SMART Amplification Kit (634859, Takara Biomedical Technology, Beijing, China). All procedures pertaining to the kit followed the provided guidelines. The mRNA transcript levels of all genes assessed were analyzed through quantitative reverse transcription polymerase chain reaction (qRT-PCR). Genes associated with proliferation, such as Oct4, Sox2, and Nanog, were identified, along with apoptosis-related genes that included Caspase3 and Bax. The synthesis of primers was carried out by Shanghai Sangon Biotech Co., Ltd. The primers were prepared at a working concentration of 10 ng/μL. The threshold cycle (Ct) values for all genes tested were normalized against the Ct value of GAPDH, with three replicates designated for every sample. The relative expression levels of the genes were determined using the 2^−∆∆Ct^ method. PCR amplification was executed under specific conditions: an initial denaturation step at 94 °C for 5 min, followed by 40 cycles comprising 30 s at 94 °C, 30 s at 59 °C, and 30 s at 72 °C. GAPDH was utilized as the reference gene during qRT-PCR, with its average expression level serving as the standard for comparison. Five independent experiments were performed for analyzing gene expression, each replicated three times. The primers utilized for qRT-PCR can be found in Table 2.

**Table 2:** List of qRT-PCR primers.

Primer	Sequence	Annealing Temperature	Product Length
Nanog	F: CCAGGGGTGTTTGGTGAACT	60 °C	211 bp
	R: TGCTCCACGTGGGGTTATTC		
Oct4	F: GGGCAAACGATCAAGCAGTG	60 °C	172 bp
	R: CTCAGGGAATGGGACCGAAG		
Sox2	F: AGAGTGTTTGCAAAAGGGGGA	60 °C	147 bp
	R: CGCCGCCGATGATTGTTATT		
Caspase3	F: AGCGTCGTAGCTGAACGTAAA	59 °C	247 bp
	R: CTGCATCCACGTCTGTACCA		
Bax	F: ACAGGGGCCCTTTTGCTTCA	60 °C	112 bp
	R: TCTCGGGGAGAGTCTGTGTC		
GAPDH	F: CTGCCCGTTCGACAGATAGC	60 °C	262 bp
	R: TGATGACGAGCTTCCCGTTC		

### 2.9. Statistical Analysis

Statistical graphs were created with GraphPad Prism software version 8.0 (GraphPad Software, Inc., San Diego, CA, USA). The data are expressed as mean ± standard deviation, derived from at least three independent experiments. A two-tailed Student’s *t*-test was utilized to compare variables among different groups. For analyzing multiple comparisons, analysis of variance (ANOVA) was performed, succeeded by pairwise comparisons using the Tukey honest significant difference (Tukey HSD) test. The fluorescence intensity for each image was measured employing ImageJ software (version 1.8.0). A *p*-value below 0.05 was considered statistically significant.

## 3. Results

### 3.1. Results of Superovulation and Embryo Recovery

**Figure 2:**
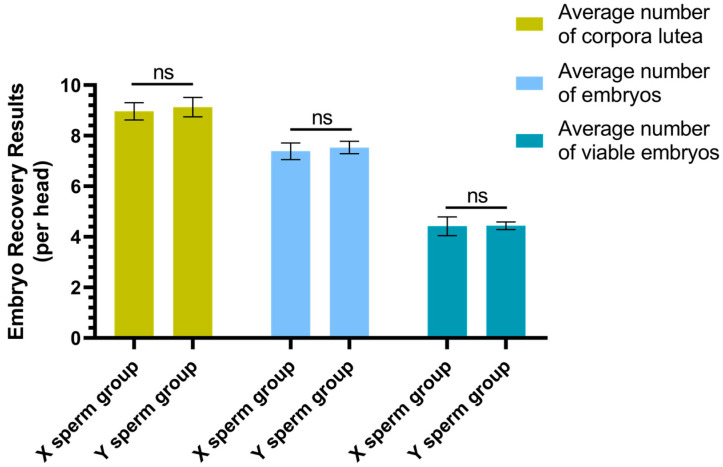
Results of Superovulation and Embryo Recovery. In the figure, “ns” indicates that there is no significant difference between the two groups (*p* ≥ 0.05).

The results of superovulation and embryo recovery are presented in Figure 2. The average number of corpora lutea per individual in the X sperm group was 8.96 ± 0.33, which did not differ significantly from that in the Y sperm group (9.13 ± 0.38) (*p* ≥ 0.05). Similarly, the average number of embryos per individual in the X sperm group was 7.38 ± 0.32, which was not significantly different from the Y sperm group (7.53 ± 0.24) (*p* ≥ 0.05). Additionally, the average number of viable embryos per individual in the X sperm group was 4.42 ± 0.37, which did not significantly differ from that in the Y sperm group (4.44 ± 0.15) (*p* ≥ 0.05).

### 3.2. Embryo Gender Identification

**Figure 3:**
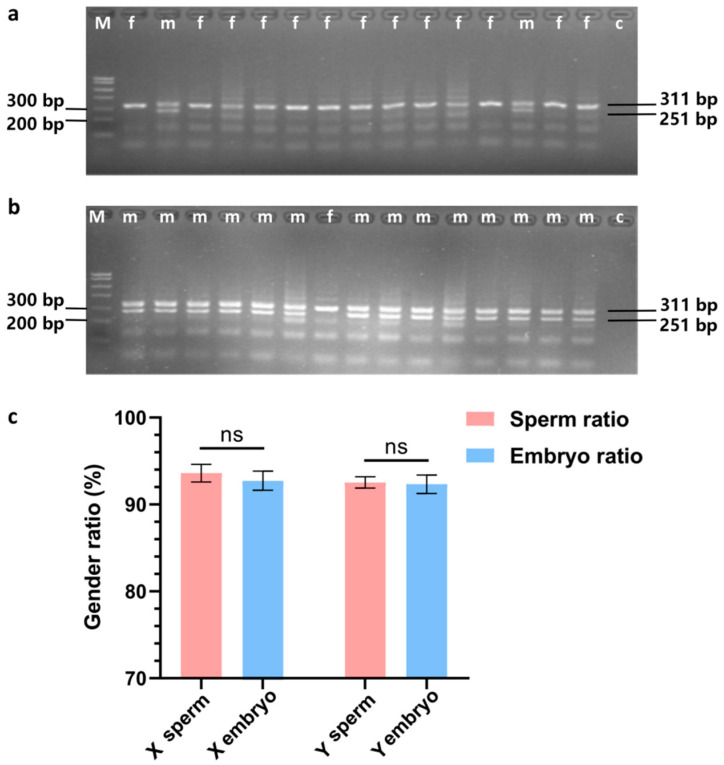
Embryo Gender Identification Result. In (**a**,**b**) “M” denotes the DNA marker (700 bp), while “c” indicates the negative control. The presence of two bands in the figures signifies the PCR results for male embryos (denoted by the letter “m”), whereas a single band represents the PCR results for female embryos (denoted by the letter “f”). In (**c**), “ns” indicates that there is no significant difference between the two groups (*p* ≥ 0.05).

The results of embryonic sex determination are presented in Figure 3. The agarose gel electrophoresis images for sex determination of embryos obtained through artificial insemination with X semen are displayed in Figure 3a, while those for embryos obtained using Y semen are shown in Figure 3b. The proportion of female embryos, determined to be 92.73 ± 1.10, did not significantly differ from the proportion of embryos derived from X semen, which was 93.60 ± 1.01 (*p* ≥ 0.05). Similarly, the proportion of male embryos, recorded at 92.33 ± 1.05, did not significantly differ from the proportion of embryos obtained from Y semen, which was 92.53 ± 0.65 (*p* ≥ 0.05), as illustrated in Figure 3c.

### 3.3. Fluorescence Staining Detection of Cell Proliferation and Apoptosis in Embryonic Cells

**Figure 4:**
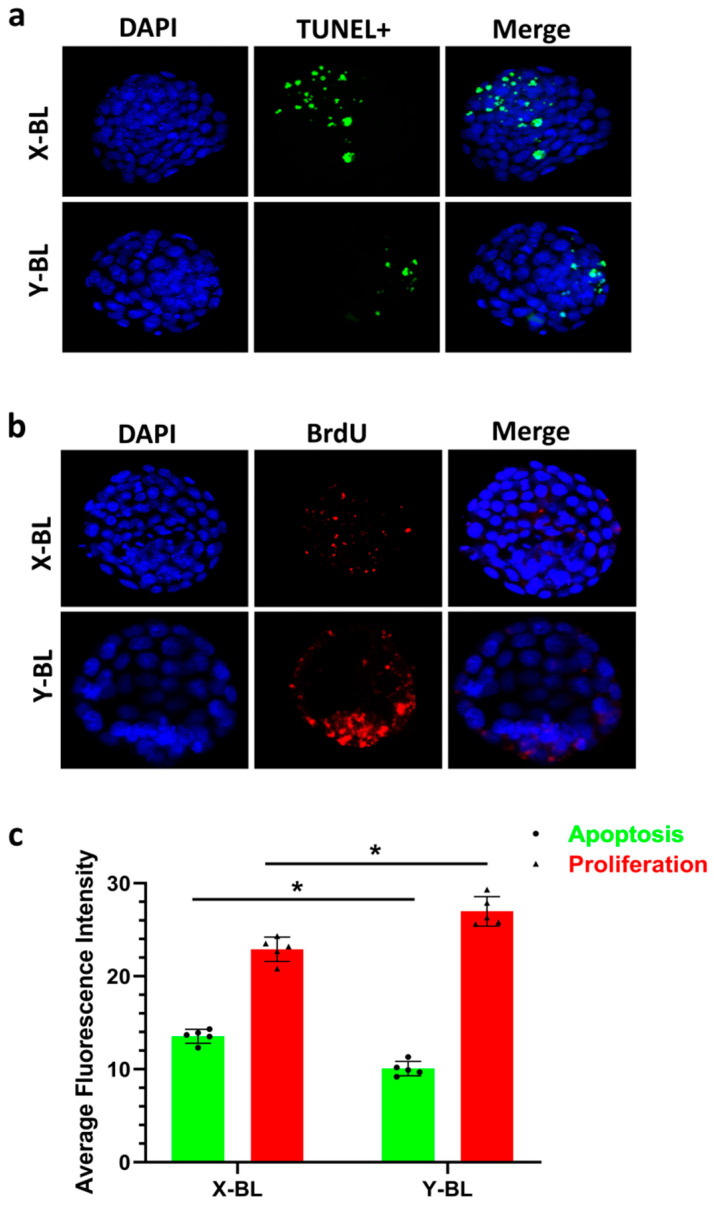
Fluorescence staining results of proliferation and apoptosis in embryonic cells. In panel (**a**), green fluorescence indicates apoptotic cells stained by TUNEL, while blue fluorescence represents DAPI staining; the original image was magnified 100×. In panel (**b**), red fluorescence denotes proliferating cells within the embryo stained by BrdU, with blue fluorescence again indicating DAPI staining; the original image was magnified 100×. In panel (**c**), an asterisk (*) signifies a statistically significant difference between the two groups (*p* < 0.05).

Apoptotic cells were stained green using the TUNEL method (Figure 4a), while proliferating cells within the embryo were stained red with BrdU (Figure 4b). Statistical analysis indicated that the fluorescence intensity of apoptotic cells in the X-BL group (13.54 ± 0.75) was significantly higher than that in the Y-BL group (10.06 ± 0.78) (*p* < 0.05). In contrast, the fluorescence intensity of proliferating cells in the X-BL group (22.90 ± 1.30) was significantly lower than that in the Y-BL group (26.98 ± 1.58) (*p* < 0.05) (Figure 4c).

### 3.4. Detection of Genes Related to Embryonic Proliferation and Apoptosis

**Figure 5:**
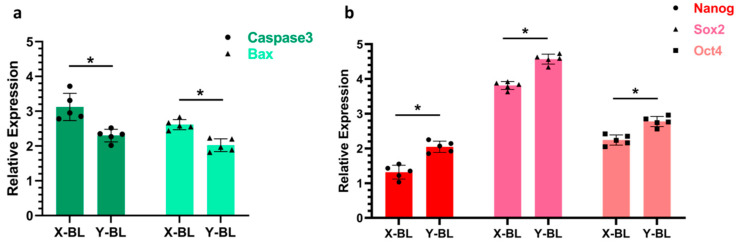
Detection results of genes related to embryonic proliferation and apoptosis. (**a**) illustrates the expression levels of apoptosis-related genes Caspase3 and Bax in the embryo, while (**b**) presents the expression levels of proliferation-related genes Nanog, Sox2, and Oct4. An asterisk (*) indicates a significant difference between the two groups (*p* < 0.05).

The expression levels of apoptosis-related genes, specifically Caspase3 and Bax, as well as proliferation-related genes, including Nanog, Sox2, and Oct4, were analyzed in embryos (Figure 5). The results indicated that the expression levels of Caspase3 and Bax in the X-BL group (3.12 ± 0.39 and 2.61 ± 0.14, respectively) were significantly higher than those in the Y-BL group (2.30 ± 0.18 and 2.02 ± 0.18, respectively) (*p* < 0.05). Conversely, the expression levels of Nanog, Sox2, and Oct4 in the X-BL group (1.31 ± 0.19, 3.81 ± 0.11, and 2.24 ± 0.14, respectively) were significantly lower than those in the Y-BL group (2.04 ± 0.16, 4.57 ± 0.14, and 2.77 ± 0.14, respectively) (*p* < 0.01).

## 4. Discussion

Previous studies have indicated that there are differences in the developmental rates between early female and male embryos; however, most of these studies were conducted using in vitro cultured embryos, whose development can be influenced by various external factors. To date, there has been limited research on the differences in developmental rates between early male and female embryos in vivo. In this study, we employed the CIDR + FSH + PGF2α + GnRH method to induce superovulation in donor cows, followed by artificial insemination with high-purity X and Y frozen semen, respectively. Seven days later, embryo flushing was performed to obtain early female and male embryos. We subsequently conducted proliferation and apoptosis assays on these early embryos. Our findings reveal that the developmental rate of early male embryos in vivo is faster than that of female embryos.

The rate of embryonic development is closely related to its quality. A core biological characteristic of rapid embryonic development lies in the significant enhancement of cell proliferation activity, and this indicator has been widely proven to serve as a key basis for evaluating the health status and developmental potential of embryos [20]. Precise regulation of cell proliferation relies on a regulatory network composed of multiple genes. Among them, Nanog, Oct4, and Sox2, as core transcription factors maintaining stem cell pluripotency, can not only regulate cell cycle progression but also play irreplaceable key roles in sustaining stem cell self-renewal capacity and inhibiting differentiation processes [21]. To investigate the impact of sex differences on the regulation of embryonic development, this study employed qRT-PCR technology to detect the relative mRNA expression levels of Nanog, SOX2, and Oct4 in male and female embryos at the blastocyst stage [22,23,24]. The experimental results showed that the expression levels of the above three core regulatory genes in male embryos were significantly higher than those in female embryos. This sex-biased expression pattern was highly consistent with the conclusions drawn by Sidrat et al. in their study on mouse embryonic development, which further verified the conservation of the regulatory mechanism of sex differences during embryonic development [13]. Nanog, Sox2, and Oct4 are essential pluripotency regulators that promote stem cell proliferation [25], and their heightened expression in male embryos may account for the observed tendency towards increased cell proliferation rates, resulting in accelerated and enhanced embryonic development. Meanwhile, the expression results of apoptosis-related genes (Caspase3 and Bax) detected synchronously in this study showed that their expression levels were significantly negatively correlated with the aforementioned pluripotency genes, i.e., the expression levels of apoptosis-related genes were lower in male embryos. This result further strengthens the conclusion of the developmental advantage of male embryos from the perspective of the balance between cell survival and proliferation. Additionally, the results of our immunofluorescence assays for proliferation and apoptosis corroborate these findings.

In summary, early-stage male embryos developed in vivo demonstrate higher quality, accelerated developmental speed, and superior developmental processes compared to their female counterparts.

## 5. Conclusions

Our research findings demonstrate that during the in vivo development of early bovine embryos, the expression levels of proliferation-related genes (Nanog, SOX2, and Oct4) are higher in male embryos than in female embryos, while the expression levels of apoptosis-related genes (Caspase3 and Bax) are lower in male embryos. Consequently, during the in vivo development of early bovine embryos, male embryos exhibit a greater number of proliferating cells, a reduced proportion of apoptotic cells, and a faster developmental rate compared to female embryos.

## Figures and Tables

**Table 1:** The primers used for nested PCR.

Primer	Sequence form 5′ to 3′	Annealing Temperature °C	Fragment Length
AMELY-1	F: CATGGTGCCAGCTCAGCAG	62	X: 349 bp
	R: CCGCTTGGTCTTGTCTGTTGC		Y: 289 bp
AMELY-2	F: CAGCAACCAATGATGCCAGTTC	62	X: 311 bp
	R: GTCTTGTCTGTTGCTGGCCA		Y: 251 bp

## Data Availability

The original contributions presented in the study are included in the article; further inquiries can be directed to the corresponding authors.
